# Causal relationship between multiple sclerosis and spinal stenosis: Two-sample Mendelian randomization

**DOI:** 10.1097/MD.0000000000039554

**Published:** 2024-09-06

**Authors:** Guang-hua Deng

**Affiliations:** a Ya’an Hospital of Traditional Chinese Medicine.

**Keywords:** Mendelian randomization, multiple sclerosis, spinal stenosis

## Abstract

To investigate the causal relationship between multiple sclerosis and spinal stenosis using Mendelian randomization (MR). Genetic loci independently associated with multiple sclerosis and spinal stenosis in people of European origin were selected as instrumental variables using pooled data from genome wide association studies (GWAS). Three MR analyses, MR-Egger, Weighted median and inverse variance weighting (IVW), were used to investigate the causal relationship between multiple sclerosis and spinal stenosis. Heterogeneity and multiplicity tests were performed, and sensitivity analyses were performed using the “leave-one-out” method to explore the robustness of the results. The IVW results showed an OR (95% CI) of 1.05 (1.01–1.08), *P* = .016, indicating a causal relationship between MS and spinal stenosis. And no heterogeneity and multiplicity were found by the test, and sensitivity analysis also showed robust results. In this study, genetic data were analyzed and explored using 2-sample MR analysis, and the results showed a causal relationship between multiple sclerosis and the occurrence of spinal stenosis.

## 1. Introduction

Spinal stenosis is a neurological disorder that manifests itself as a narrowing of the space within the spinal canal, resulting in compression of the nerve roots or spinal cord.^[1,2]^ This disease is common in the elderly, especially those over 50 years of age.^[3]^ Spinal stenosis can affect a patient’s quality of life, and in severe cases it can even lead to limited mobility. The causes of spinal stenosis are varied and include degenerative changes, bone spurs, and ligamentous hyperplasia. These factors lead to narrowing of the space within the spinal canal, which compresses the nerve roots or spinal cord.^[4,5]^ Symptoms mainly include low back pain, radiating pain in the lower limbs, sensory abnormalities, and loss of muscle strength.^[6,7]^ Severe spinal stenosis may also cause dysfunction in urination and defecation.^[8]^ Multiple sclerosis is a chronic, autoimmune disease that primarily affects the central nervous system.^[9,10]^ Patients may experience a variety of neurological symptoms, such as muscle weakness, sensory abnormalities, balance problems, coordination disorders, and vision problems.^[11–13]^ These symptoms may affect patients’ daily activities and quality of life. Some studies have suggested that patients with multiple sclerosis may be more prone to spinal stenosis, but relevant studies are lacking.^[14]^ Therefore, the causal relationship between multiple sclerosis and spinal stenosis still needs further investigation.

The association between multiple sclerosis and spinal stenosis may be influenced to some extent by confounding factors and reverse causality inherent in traditional observational studies.^[15]^ In contrast, Mendelian randomization (MR), a genetic epidemiological method, is a useful tool for assessing the causal role of multiple sclerosis and spinal stenosis.^[16]^ By using genetic variants such as single nucleotide polymorphisms (SNPs) as instrumental variants that can modify disease risk factors or exposures, MR studies can enhance causal inference of exposure-outcome associations.^[17]^ According to Mendel’s laws of inheritance, genetic variants are not susceptible to confounding factors because they are randomly assigned during gamete formation.^[18]^ In addition, confounders and reverse causality can be minimized as genotypes cannot change as the disease progresses.^[19]^

To this end, we conducted a 2-sample MR study to examine the causal relationship between multiple sclerosis and spinal stenosis. We aimed to provide significant evidence for the causal role of MS in causing spinal stenosis.

## 2. Data and methods

### 2.1. Data sources

The genome wide association studies (GWAS) data for multiple sclerosis and spinal stenosis were obtained via the IEU OpenGWAS project (mr cieu.ac.uk) website. The website was accessed on 2023-08-06.The population source for all final data was European, male and female. Including multiple sclerosis (ebi-a-GCST005531) containing 132,089 SNPs with a sample size of 38,582, and spinal stenosis (finn-b-M13_SPINSTENOSIS) containing 16,380,277 SNPs with 9169 in the experimental group and 164,682 in the control group. This study was a re-analysis of previously collected and published public data and therefore did not require additional ethical approval.

### 2.2. Conditioning of SNP as an instrumental variable

Instrumental variables were highly correlated with exposure, with *F* > 10 as a strong correlation criterion.^[20]^ Instrumental variables are not directly correlated with the outcome and only affect the outcome through exposure, i.e. there is no genetic pleiotropy. In this study, the nonexistence of genetic pleiotropy was indicated by the non-zero intercept term of the MR-Egger regression model (*P* < .05).^[21]^ Instrumental variables were not associated with untested confounding.^[22]^ The human genotype-phenotype association database Phenoscanner V2 was searched for phenotypes associated with the instrumental variables at the genome-wide significance level to determine whether these SNPs were associated with potential risk factors.^[23]^

### 2.3. SNP screening

Significant SNPs were screened from the GWAS pooled data for multiple sclerosis (with *P* < 5 × 10^−8^ as the screening condition)^[24]^; the chain imbalance coefficient *r*^2^ was set to 0.001 and the width of the chain imbalance region to 10,000 kb to ensure that individual SNPs were independent of each other.^[25]^ The multiple sclerosis-associated SNPs screened above were extracted from the GWAS pooled data of spinal stenosis, while SNPs directly associated with outcome indicators were excluded (*P* < 5 × 10^−8^). The *F* value of each SNP was calculated, and SNPs with weak instrumental variables (*F* value < 10) were excluded.^[26]^ And the human genotype-phenotype association database was queried to screen for potentially relevant risk factor SNPs and exclude them.^[27]^

### 2.4. Causality validation methods

The causal relationship between exposure (multiple sclerosis) and outcome (spinal stenosis) was mainly verified using inverse variance weighted (IVW) as, supplemented by 3 MR analysis methods, MR-Egger and weighted median, with SNPs as instrumental variables.

### 2.5. Sensitivity analysis

Sensitivity analyses were performed using several methods. First, the Cochran *Q* test was used to assess the heterogeneity between the individual SNP estimates, and a statistically significant Cochran *Q* test demonstrated significant heterogeneity in the analyses. Second, Mendelian randomization pleiotropy residual sum and outlier (MR PRESSO) was used to validate the results in the IVW model, to correct for the effect of outliers, and if outliers existed, they were removed and the analysis was repeated. Third, the horizontal multiplicity of SNPs was tested using the MR Egger intercept test (MR Egger intercept test), and if the intercept term in the MR Egger intercept test analysis was statistically significant, it indicated that the MR analysis had significant horizontal multiplicity. Fourth, “leave-one-out” sensitivity analyses were performed by removing a single SNP at a time to assess whether the variant drove the association between the exposure and outcome variables. Fifth, funnel plots and forest plots were constructed to visualize the results of the sensitivity analyses. *P* < .05 suggests that there is a potential causal relationship in the MR analyses, which is statistically significant. All statistical analyses were performed using the “TwoSampleMR” package in R software version 4.3.0.

## 3. Results

### 3.1. Instrumental variables

Forty-six SNPs that were strongly associated with multiple sclerosis (*P* < 5 × 10^−8^) without chain imbalance (*r*^2^ < 0.001, kb = 10,000) were screened in the present study. Forty-six SNPs were left by taking the intersection with SNPs in the pooled data from the GWAS for spinal stenosis, and also by eliminating SNPs that were directly associated with the outcome metrics. In our study, the *F* values of each SNP were all >10, indicating no weak instrumental variables (see Table 1 for details). We searched the human genotype-phenotype association database and found no potentially relevant risk factor SNPs.

**Table 1:** Information on the final screening of multiple sclerosis SNPs from GWAS data (n = 46).

ID	SNP	Effect_Allele	Other_Allele	β	SE	*P*	F
1	rs1014486	C	T	0.10075	1.16E-09	0.0165552	37
2	rs1021156	C	T	−0.115113	5.60E-10	0.0185627	38
3	rs10420809	T	C	0.121377	7.96E-09	0.0210384	33
4	rs1077667	T	C	−0.15187	3.54E-13	0.020884	52
5	rs10892299	T	C	−0.115074	4.03E-08	0.0209625	30
6	rs11052877	G	A	0.0998453	5.37E-09	0.0171106	34
7	rs11154801	A	C	0.102557	2.35E-09	0.0171742	35
8	rs11172342	T	G	−0.121377	9.46E-12	0.0178116	46
9	rs11554159	A	G	−0.141448	2.58E-13	0.0193376	53
10	rs11865086	A	C	−0.0935417	1.77E-08	0.0166057	31
11	rs12087340	T	C	0.19721	5.13E-12	0.0285732	47
12	rs12927355	T	C	−0.193099	8.19E-27	0.0180128	114
13	rs1323292	A	G	0.160404	3.68E-13	0.0220734	52
14	rs13426106	A	G	0.154436	1.67E-13	0.0209466	54
15	rs17066096	G	A	0.131028	5.91E-12	0.0190398	47
16	rs1800693	C	T	0.134531	6.92E-16	0.0166665	65
17	rs1813375	T	G	0.143234	5.75E-18	0.016583	74
18	rs2104286	C	T	−0.18983	7.61E-23	0.0192926	96
19	rs212407	A	G	0.138917	2.56E-15	0.0175607	62
20	rs2364482	G	T	0.124869	1.40E-09	0.0206208	36
21	rs3115627	G	A	−0.313342	1.77E-67	0.0180536	301
22	rs3130283	C	A	−0.82549	1.00E-200	0.0216086	1459
23	rs34383631	T	C	0.105261	5.69E-10	0.0169808	38
24	rs3748817	C	T	−0.129312	1.33E-12	0.0182356	50
25	rs41286801	T	C	0.181488	7.92E-16	0.0225299	64
26	rs4410871	C	T	0.112385	1.98E-09	0.0187326	35
27	rs4780355	C	T	−0.100705	3.47E-08	0.0182572	30
28	rs4796791	C	T	−0.0962189	1.81E-08	0.0170927	31
29	rs4944958	G	A	−0.107059	8.69E-09	0.0186044	33
30	rs4947337	C	T	−0.414153	1.34E-21	0.0433821	91
31	rs4976646	C	T	0.123102	1.04E-12	0.0172772	50
32	rs57271503	A	G	−0.172213	4.11E-15	0.0219337	61
33	rs60600003	G	T	0.149282	2.53E-08	0.0267953	31
34	rs6677309	C	A	−0.292628	1.45E-28	0.0263937	122
35	rs67297943	C	T	−0.11149	4.83E-08	0.020429	29
36	rs6881706	T	G	−0.110596	4.87E-09	0.0189003	34
37	rs706015	G	T	0.129272	1.29E-09	0.0213017	36
38	rs71624119	A	G	−0.116871	2.70E-09	0.0196459	35
39	rs74796499	A	C	−0.270759	8.47E-11	0.0417066	42
40	rs7783	G	A	0.0989399	9.56E-09	0.0172419	32
41	rs7923837	A	G	−0.100705	4.58E-09	0.01718	34
42	rs8070345	C	T	−0.134531	5.43E-16	0.0166057	65
43	rs842639	A	G	0.107919	1.70E-09	0.0179144	36
44	rs9277641	A	G	−0.219899	1.19E-24	0.0214547	105
45	rs9282641	A	G	−0.193949	1.74E-10	0.0303867	40
46	rs9967792	C	T	0.102587	1.80E-09	0.0170554	36

GWAS = genome-wide association study, SE, SNP = single nucleotide polymorphism.

### 3.2. Causal relationship between multiple sclerosis and spinal stenosis

**Figure 1.**
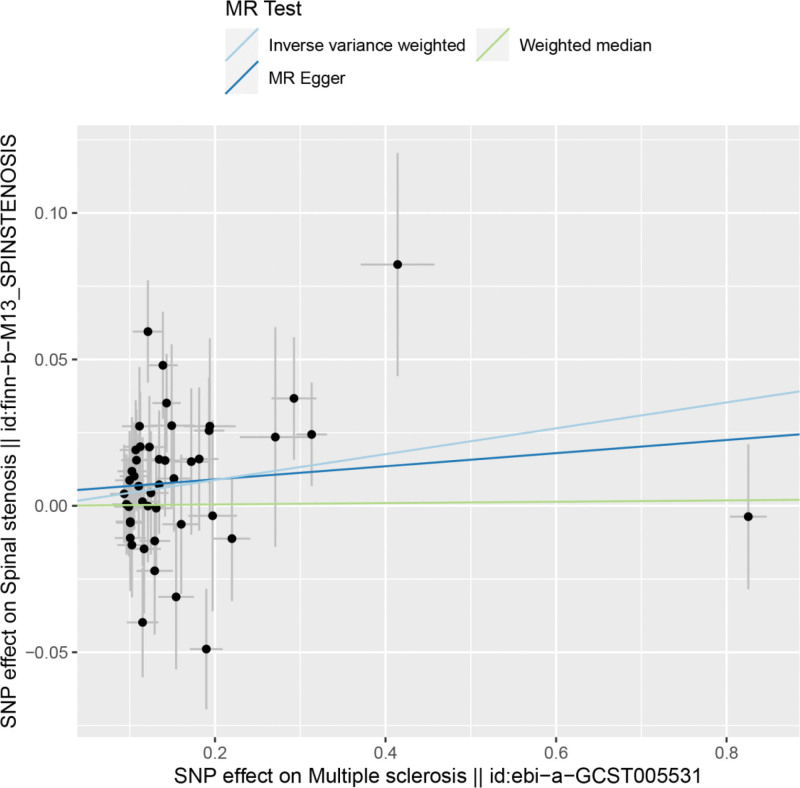
Scatter plot of multiple sclerosis and spinal stenosis. MR = Mendelian randomization, SNP = single nucleotide polymorphism.

**Figure 2.**
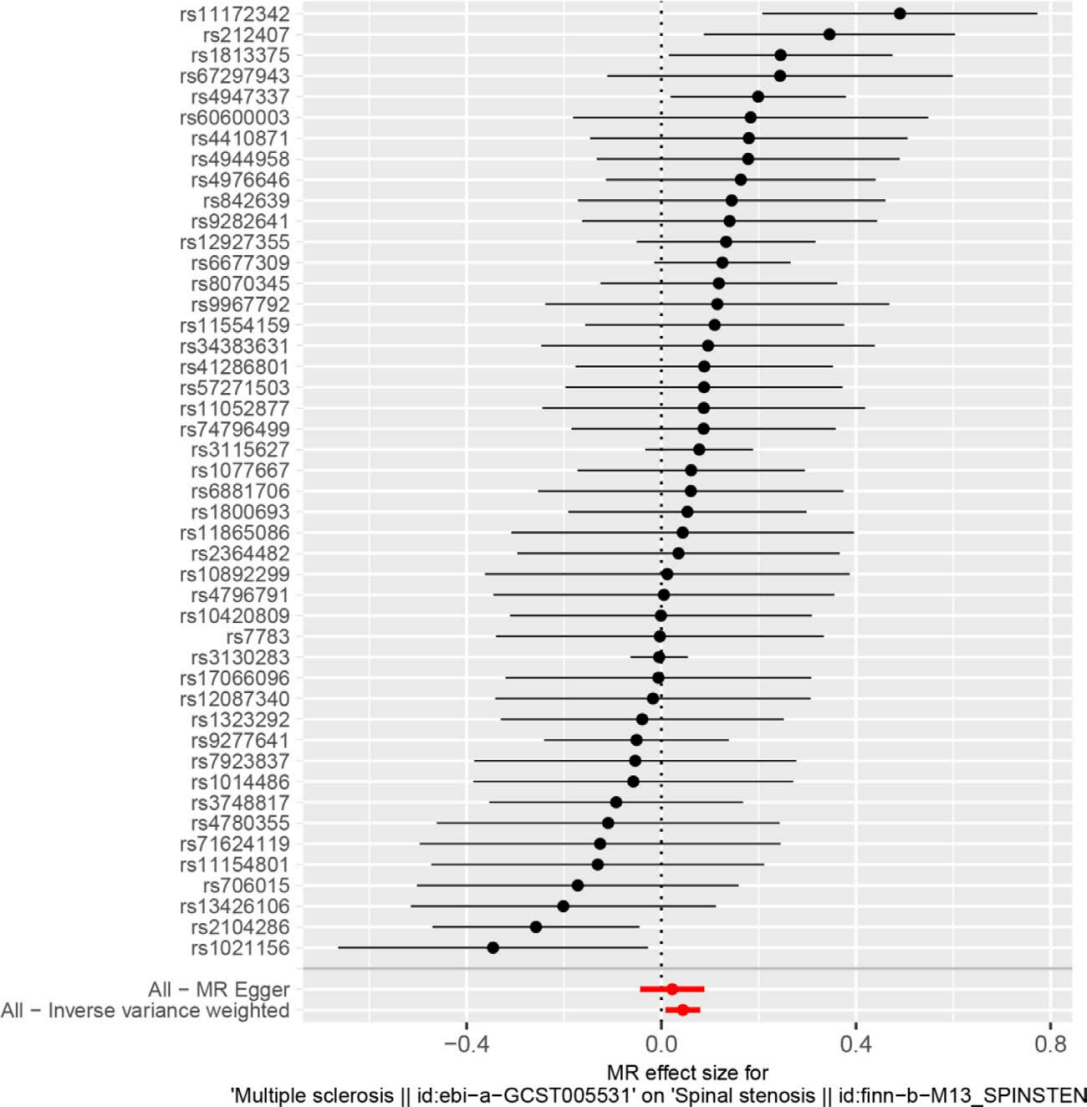
Forest plot of multiple sclerosis and spinal stenosis. MR = Mendelian randomization.

By MR analysis, the results of IVW showed a positive correlation between multiple sclerosis and spinal stenosis, and the differences were all statistically significant, i.e., there was a causal relationship between multiple sclerosis and spinal stenosis. IVW:OR = 1.05, 95% CI = 1.01–1.08, *P* = .016; weighted median:OR = 1.00, 95% CI = 0.95–1.05, *P* = .928; MR Egger:OR = 1.02, 95% CI = 0.96–1.09, *P* = .509 (see Table 2 for details). We can see from both the scatter plot (Fig. 1) and the forest plot (Fig. 2) that having MS increases the risk of developing spinal stenosis.

**Table 2:** MR regression results of the 3 methods.

Method	β	SE	OR (95% CI)	*P*
IVW	0.044	0.018	1.05 (1.01–1.08)	.016
WME	0.002	0.025	1.00 (0.95–1.05)	.928
MR-Egger	0.022	0.034	1.02 (0.96–1.09)	.509

CI = confidence interval, IVW = inverse variance weighting, MR =Mendelian randomization, OR = odds ratio, SE, WME = weighted median.

### 3.3. Sensitivity analysis

**Figure 3.**
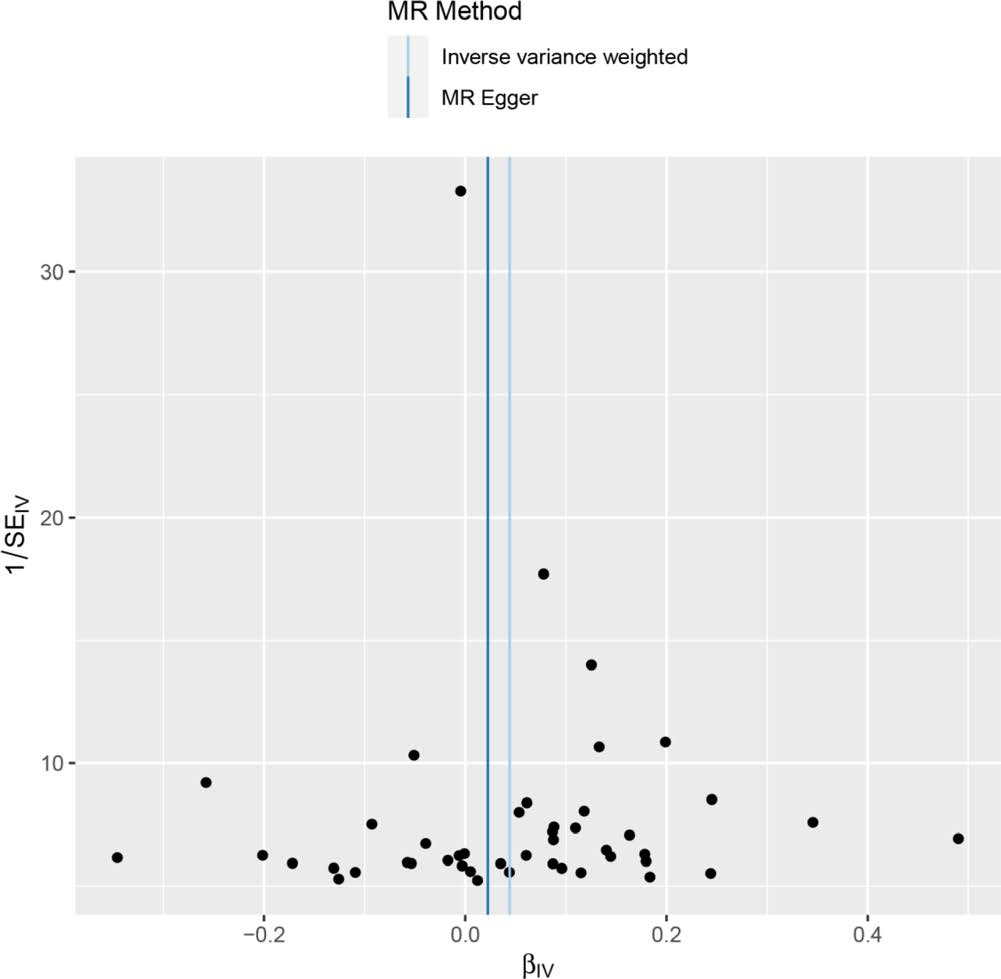
Funnel plot of multiple sclerosis and spinal stenosis. MR = Mendelian randomization, SE.

**Figure 4.**
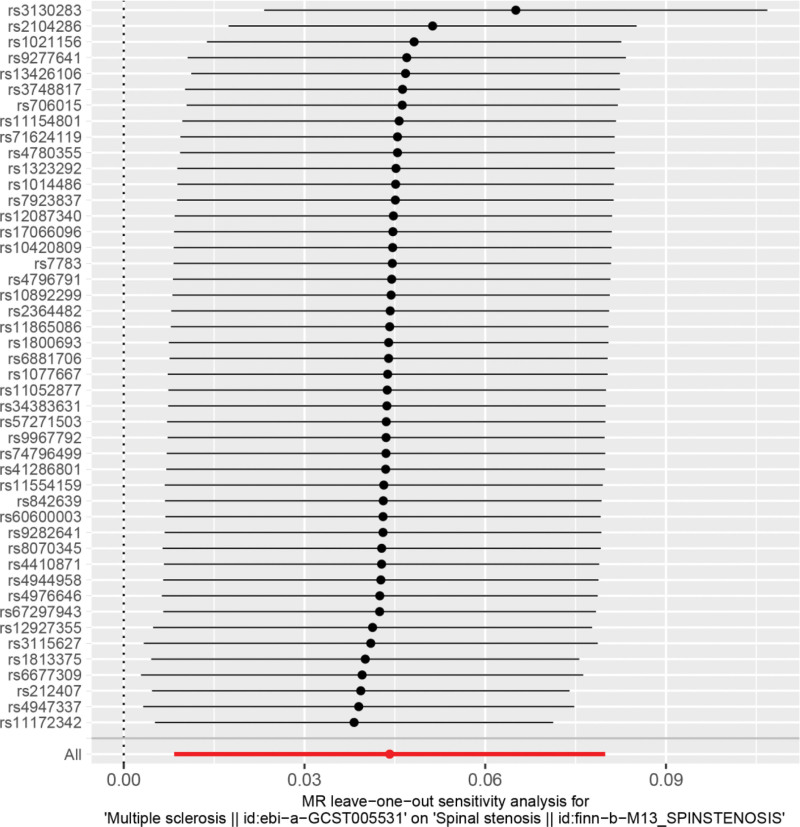
Analysis of multiple sclerosis and spinal stenosis by the leave-one-out method. MR = Mendelian randomization.

Heterogeneity was tested using the IVW method (Cochran *Q* test, *P* = .143), and the results suggested that there was no heterogeneity. A funnel plot was drawn to show the heterogeneity results, as shown in Figure 3. MR-PRESSO was used to screen for SNPs that could lead to heterogeneity, and the results did not reveal any SNPs that would lead to heterogeneity in the results. The result of Global test by MR-PRESSO suggested that there was no pleiotropy (*P* = .448). The “leave-one-out” method uses the IVW method by default, and as can be seen in Figure 4, no single SNP will have a large impact on the overall results after eliminating any SNP, indicating that the results are robust.

## 4. Discussion

It is known that multiple sclerosis may be an observational risk factor for spinal stenosis, but the causality of this association is unclear. Our MR study aimed to reveal the causal relationship between multiple sclerosis and spinal stenosis. The results showed a causal relationship between multiple sclerosis and the occurrence of spinal stenosis, as demonstrated by the 2-sample MR results, with an OR (95% CI) of 1.05 (1.01–1.08), *P* = .016.

Young et al^[14]^ found that the probability of suffering from spinal stenosis in the population of patients with multiple sclerosis was much higher than in the general population.

The present study confirms the causal relationship between multiple sclerosis and with the occurrence of spinal stenosis from a genetic point of view. The results of this study are consistent with Young’s conclusion that multiple sclerosis is a risk factor for the development of spinal stenosis and that having multiple sclerosis increases the incidence of spinal stenosis.

The present study used a Mendelian randomization study rather than a retrospective study, which is susceptible to confounding factors and reverse causation, and therefore the causal inferences obtained are considered to be of limited value. In contrast, Mendelian randomization (MR) analysis is a new epidemiological approach that uses genetic variation as an instrumental variable of exposure to enhance causal inference. This approach reduces the effects caused by confounding factors.^[28]^

At the same time this study has some limitations. Firstly, as all the data are from people of European origin, the results are not representative of a truly random population sample, nor are they applicable to other so races. Secondly, although various sensitivity analyses have been performed in this study to test the hypotheses of the MR study, it is difficult to completely rule out horizontal pleiotropy of instrumental variables. Finally, the current sample size of GWAS data is still not large enough, and more in-depth studies using more GWAS data are needed in the future.

## 5. Conclusion

In conclusion, this study used 2-sample MR analysis to analyze and explore the genetic data, and the results showed a causal relationship between multiple sclerosis and the occurrence of spinal stenosis.

## Author contributions

**Conceptualization:** Gaung-hua Deng.

**Data curation:** Gaung-hua Deng.

**Formal analysis:** Gaung-hua Deng.

**Funding acquisition:** Gaung-hua Deng.

**Investigation:** Gaung-hua Deng.

**Methodology:** Gaung-hua Deng

**Project administration:** Gaung-hua Deng.

**Resources:** Gaung-hua Deng.

**Software:** Gaung-hua Deng.

**Supervision:** Gaung-hua Deng.

**Validation:** Gaung-hua Deng.

**Visualization:** Gaung-hua Deng.

**Writing – original draft:** Gaung-hua Deng.

**Writing – review & editing:** Gaung-hua Deng.

## References

[1] Rhon D. Lumbar spinal stenosis. N Engl J Med. 2008;358:2647; author reply 2647-8. 18557179

[2] Walter KL O’Toole JE. Lumbar spinal stenosis. JAMA. 2022;328:310. 35503646 10.1001/jama.2022.6137

[3] Karlsson T Försth P Skorpil M. Decompression alone or decompression with fusion for lumbar spinal stenosis: a randomized clinical trial with two-year MRI follow-up. Bone Joint J. 2022;104-B:1343–51. 36453045 10.1302/0301-620X.104B12.BJJ-2022-0340.R1 PMC9680197

[4] Kreiner DS Shaffer WO Baisden JL. North American Spine Society. An evidence-based clinical guideline for the diagnosis and treatment of degenerative lumbar spinal stenosis (update). Spine J. 2013;13:734–43. 23830297 10.1016/j.spinee.2012.11.059

[5] Heary RF Anderson PA Arnold PM. Introduction. Lumbar spinal stenosis. Neurosurg Focus. 2019;46:E1. 10.3171/2019.2.FOCUS19151 31042650

[6] Bai Q Wang Y Zhai J Wu J Zhang Y Zhao Y. Current understanding of tandem spinal stenosis: epidemiology, diagnosis, and surgical strategy. EFORT Open Rev. 2022;7:587–98. 35924651 10.1530/EOR-22-0016 PMC9458946

[7] Schizas C. Reviewer’s Comment to“The association between preoperative MRI findings and clinical improvement in patients included in the NORDSTEN spinal stenosis trial” by J. Aaen *et al*. (Eur Spine J [2022]; doi: 10.1007/s00586-022-07317-5): does the severity of radiological stenosis influence post-operative results following spinal decompression? Eur Spine J. 2022;31:2786–7. 35982346 10.1007/s00586-022-07350-4

[8] Wang AY Patel J Kanter M. The emerging significance of amyloid deposits in the ligamentum flavum of spinal stenosis patients: a review. World Neurosurg. 2023;177:88–97. 37331471 10.1016/j.wneu.2023.06.037

[9] Thompson AJ Baranzini SE Geurts J Hemmer B Ciccarelli O. Multiple sclerosis. Lancet. 2018;391:1622–36. 29576504 10.1016/S0140-6736(18)30481-1

[10] Compston A Coles A. Multiple sclerosis. Lancet. 2008;372:1502–17. 18970977 10.1016/S0140-6736(08)61620-7

[11] Reich DS Lucchinetti CF Calabresi PA. Multiple sclerosis. N Engl J Med. 2018;378:169–80. 29320652 10.1056/NEJMra1401483 PMC6942519

[12] Multiple sclerosis. Nat Rev Dis Primers. 2018;4:44. 30410088 10.1038/s41572-018-0046-z

[13] Lin M Zhang J Zhang Y Luo J Shi S. Ocrelizumab for multiple sclerosis. Cochrane Database Syst Rev. 2022;5:CD013247. 35583174 10.1002/14651858.CD013247.pub2 PMC9115862

[14] Young WF Weaver M Mishra B. Surgical outcome in patients with coexisting multiple sclerosis and spondylosis. Acta Neurol Scand. 1999;100:84–7. 10442447 10.1111/j.1600-0404.1999.tb01042.x

[15] Estimating dose-response relationships for vitamin D with coronary heart disease, stroke, and all-cause mortality: observational and Mendelian randomisation analyses. Lancet Diabetes Endocrinol. 2021;9:837–46. 34717822 10.1016/S2213-8587(21)00263-1 PMC8600124

[16] Emdin CA Khera AV Kathiresan S. Mendelian randomization. JAMA. 2017;318:1925–6. 29164242 10.1001/jama.2017.17219

[17] Lin BD Li Y Luykx J. Mendelian randomization concerns. JAMA Psychiatry. 2018;75:407. 10.1001/jamapsychiatry.2018.0035 29516079

[18] Larsson SC Burgess S Michaëlsson K. Association of genetic variants related to serum calcium levels with coronary artery disease and myocardial infarction. JAMA. 2017;318:371–80. 28742912 10.1001/jama.2017.8981 PMC5817597

[19] Hartwig FP Borges MC Bowden J. Mendelian randomization concerns-reply. JAMA Psychiatry. 2018;75:407–8. 10.1001/jamapsychiatry.2017.4725 29516088

[20] Lieske JC. Mendelian randomization: a powerful tool to illuminate pathophysiologic mechanisms. Mayo Clin Proc. 2023;98:500–1. 37019509 10.1016/j.mayocp.2023.02.015 PMC10336724

[21] Burgess S Thompson SG. Interpreting findings from mendelian randomization using the MR-egger method. Eur J Epidemiol. 2017;32:377–89. 28527048 10.1007/s10654-017-0255-x PMC5506233

[22] Holmes MV Davey Smith G. Can mendelian randomization shift into reverse gear? Clin Chem. 2019;65:363–6. 30692117 10.1373/clinchem.2018.296806

[23] Yavorska OO Burgess S. Mendelian randomization: an R package for performing mendelian randomization analyses using summarized data. Int J Epidemiol. 2017;46:1734–9. 28398548 10.1093/ije/dyx034 PMC5510723

[24] Wu X Zhang W Zhao X. Investigating the relationship between depression and breast cancer: observational and genetic analyses. BMC Med. 2023;21:170. 37143087 10.1186/s12916-023-02876-w PMC10161423

[25] Wang J Tang H Duan Y Yang S An J. Association between sleep traits and lung cancer: a mendelian randomization study. J Immunol Res. 2021;2021:1893882. 34239941 10.1155/2021/1893882 PMC8238591

[26] Pierce BL Ahsan H Vanderweele TJ. Power and instrument strength requirements for mendelian randomization studies using multiple genetic variants. Int J Epidemiol. 2011;40:740–52. 20813862 10.1093/ije/dyq151 PMC3147064

[27] Freuer D Linseisen J Meisinger C. Association between inflammatory bowel disease and both psoriasis and psoriatic arthritis: a bidirectional 2-sample mendelian randomization study. JAMA Dermatol. 2022;158:1262–8. 36103169 10.1001/jamadermatol.2022.3682 PMC9475439

[28] Skrivankova VW Richmond RC Woolf BAR. Strengthening the reporting of observational studies in epidemiology using mendelian randomisation (STROBE-MR): explanation and elaboration. BMJ. 2021;375:n2233. 34702754 10.1136/bmj.n2233 PMC8546498

